# Remnant lipoproteins play an important role of in-stent restenosis in type 2 diabetes undergoing percutaneous coronary intervention: a single-centre observational cohort study

**DOI:** 10.1186/s12933-019-0819-z

**Published:** 2019-01-28

**Authors:** Zheng Qin, Kuo Zhou, Yue-ping Li, Jian-long Wang, Wan-jun Cheng, Cheng-ping Hu, Chao Shi, Hua He, Yu-jie Zhou

**Affiliations:** 1 Department of Cardiology, Beijing Anzhen Hospital, Capital Medical University, Beijing, 100029 China; 2 Department of Emergency Cardiology, Beijing Anzhen Hospital, Capital Medical University, Beijing, 100029 China; 3 Department of Cardiology, Beijing Anzhen Hospital, Capital Medical University, Beijing Institute of Heart Lung and Blood Vessel Disease, Beijing Key Laboratory of Precision Medicine of Coronary Atherosclerotlic Disease, Clinical Centre for Coronary Heart Disease, No. 2 Anzhen Road, Chaoyang District, Beijing, 100029 China

**Keywords:** Remnant lipoproteins, Remnant-like particle cholesterol, Type 2 diabetes mellitus, In-stent restenosis, Percutaneous coronary intervention, Very-low-density lipoprotein cholesterol

## Abstract

**Background:**

Increasing evidence has suggested that the presence of remnant lipoproteins is a significant risk factor for atherosclerosis. Remnant lipoproteins are lipoproteins that are rich in triglycerides (TGs), and the main components include very-low-density lipoprotein (VLDL) in the fasting state. Diabetic patients often have hypertriglyceridemia with elevated levels of VLDL cholesterol but normal levels of low-density lipoprotein cholesterol (LDL-C). The aim of the present study was to elucidate the potential role of remnant lipoproteins-induced atherosclerosis in the occurrence and development of in-stent restenosis (ISR) in diabetic patients with coronary artery disease.

**Methods:**

The present study enrolled 2312 patients with type 2 diabetes mellitus who underwent percutaneous coronary intervention from January 2013 to December 2014 and who were followed up by angiography. Patients were divided into two groups based on the presence or absence of ISR, and multivariate Cox’s proportional hazards regression modelling showed that remnant-like particle cholesterol (RLP-C) was an independent risk factor for ISR. According to the receiver operating characteristic curve, the optimal cutoff point of the RLP-C was identified, and the patients were further divided into 2 groups. Propensity score matching analysis was performed, and 762 pairs were successfully matched. Log-rank tests were used to compare Kaplan–Meier curves for overall follow-up to assess ISR.

**Results:**

The multivariate Cox’s proportional hazards regression analysis showed that RLP-C was independently associated with ISR, and the baseline RLP-C level at 0.505 mmol/L was identified as the optimal cutoff point to predict ISR. Patients were divided into 2 groups by RLP levels. After propensity score matching analysis, a total of 762 pairs matched patients were generated. Kaplan–Meier curves showed that the estimated cumulative rate of ISR was significantly higher in patients with RLP-C levels ≥ 0.505 mmol/L (log-rank P < 0.001; HR equal to 4.175, 95% CI = 3.045–5.723, P < 0.001) compared to patients with RLP-C levels < 0.505 mmol/L.

**Conclusions:**

The present study emphasized the importance of remnant-like particle cholesterol in cardiovascular pathology in diabetic patients. Physicians should take measures to control RLP-C below the level of 0.505 mmol/L to better prevent of in-stent restenosis in diabetic patients.

## Background

In-stent restenosis is an important factor for successful underwent percutaneous coronary intervention (PCI). Several large-scale clinical trials have confirmed that the occurrence rate of n-stent restenosis (ISR) ranges from 3 to 20% after coronary stenting implantation, even in the drug-eluting stents (DES) era [1]. Patients with diabetes mellitus (DM) have a 2–4 times higher risk of developing ISR after PCI compared with non-diabetic patients [2, 3]. The poor prognosis [4, 5] of this particular population deserves additional attention.

The high rate of ISR may be related to dyslipidaemia in DM. A recent multicentre study has shown that the prevalence of dyslipidaemia has reached up to 67.1% among diabetic patients in China [6] and is uniquely manifested by high levels of triglycerides (TGs) and very-low-density lipoprotein cholesterol (VLDL-C) but normal levels of low-density lipoprotein cholesterol (LDL-C) [7]. Remnant lipoproteins are lipoproteins that are rich in triglycerides (TGs), and the main components include VLDL in the fasting state [8]. Therefore, diabetic patients have abnormal lipid metabolism mainly due to increased remnant-like particle cholesterol (RLP-C). Recent studies have shown that high levels of remnant lipoproteins can predict coronary events in diabetic patients independent of the degree of coronary stenosis, age, gender, hypercholesterolaemia, low-density lipoprotein, hypertriglyceridaemia and other risk factors [9]. Therefore, it is of great significance to elucidate the role of RLP-induced atherosclerosis in diabetic patients.

## Methods

### Study patients

The present study is a retrospective cohort study of 2701 coronary artery disease (CAD) patients with Type 2 diabetes mellitus (T2DM) who underwent successful coronary second-generation drug-eluting stents (G2-DESs) implantation at Beijing Anzhen Hospital (Beijing, China) from January 2013 to December 2014 and were followed up by angiography. Patients who died in the hospital after baseline PCI or without sufficient clinical and angiographic data at baseline and follow up were excluded. Of these patients, 2312 patients who met the inclusion and exclusion criteria were analysed in the present study. Multivariate Cox’s proportional hazards regression modelling showed that RLP-C was an independent risk factor for ISR. According to the receiver operating characteristic (ROC), the optimal cutoff point of the RLP-C was identified, and the patients were divided into the following 2 groups: low RLP-C group (n = 1072) and high RLP-C group (n = 1240). Propensity score matching analysis was performed in the two groups with a proportion of 1:1, including baseline data (age, gender, BMI, duration of diabetes mellitus, symptom-driven hospitalization and SYNTAX score). Finally, 762 pairs of DM patients were successfully matched. Log-rank tests were used to compare Kaplan–Meier curves for overall follow-up to assess ISR between the two groups.

### Stent implantation

All enrolled patients received G2-DESs implantations in the catheterization centre. The type of G2-DESs included zotarolimus-eluting stents (Endeavor and Endeavor Resolute; Medtronic Vascular, USA), domestic sirolimus-eluting stents (Firebird2; MicroPort Medical, China), everolimus-eluting stents (Xience V and Xience Prime; Abbott Vascular, USA, Promus and Promus Element; Boston Scientific, USA). Stent implantation was performed according to current practice guidelines, and stents were selected by experienced interventional cardiologists. During the procedure, patients received a bolus of 100 IU/kg heparin with a repeated bolus of 2000 IU heparin to maintain the activated clotting time of ≥ 300 s. All patients received aspirin (100 mg/day was administered) and clopidogrel (300 mg loading dose followed by 75 mg/day for at least 12 months). When ISR was diagnosed, patients were treated with re-DES implantation. Procedural success was defined as follows: reduction of stenosis to less than 10% residual narrowing; thrombolysis in myocardial infarction (TIMI) flow grade III; improvement in ischaemic symptoms; and no major procedure related complications [7].

### Data collection

A standard case report form (CRF) was used to collect patients’ demographic and clinical characteristics, including age, gender, smoking, drinking, CAD risk factors, family history, life style, medical history and coronary angiographic information at baseline PCI and follow-up angiography. During a physical examination, anthropometric indices, such as weight, height and blood pressure (BP), were measured. Body mass index (BMI) was calculated as weight in kilograms divided by the square of the height in metres.

Coronary angiogram data, such as minimal stent diameter, average stent length and stenosis percent, were also recorded by two experienced investigators at baseline and follow-up for coronary angiography analysis.

### Laboratory analysis

Venous blood samples were collected after an overnight fast for testing lipid profiles, HbA1c, fasting blood glucose (FBG), high-sensitivity C-reactive protein (hs-CRP) and uric acid (UA) levels using standard laboratory methods at baseline PCI and follow-up angiography.

The HbA1c was tested using ion exchange high-performance liquid chromatograph (HPLC) method. Blood samples for lipid profiles were collected from patients taking statin for more than 2 weeks. The total cholesterol (TC), TG, FBG and UA levels were determined according to enzymatic methods. LDL-C and high-density lipoprotein cholesterol (HDL-C) levels were measured by homogeneous assays. RLP-C levels were calculated as TC minus LDL-C and HDL-C according to the recommendation of dyslipidaemia guidelines [10, 11].

### Disease definitions

The primary end point of the present study was the occurrence of ISR. ISR was defined as a diameter stenosis of ≥ 50% occurring in the segment inside the stent, 5 mm proximal to the stent or 5 mm distal to the stent at follow-up angiography [12]. The target lesion was considered as the most severe narrowing vessel identified by angiographic appearance with electrocardiograph (ECG) changes. Multivessel disease (MVD) was defined as a diameter stenosis of ≥ 50% occurring in 2 or more vessels.

Diabetes mellitus was defined as either a previous diagnosis of DM (treated with diet, oral agents or insulin) or a new diagnosis of DM (FBG ≥ 7.0 mmol/L on 2 occasions during hospitalization) [13]. Hypertension was defined by systolic blood pressure (SBP) ≥ 140 mmHg, diastolic blood pressure (DBP) ≥ 90 mmHg and/or the use of antihypertensive treatment in the past 2 weeks [14]. The severity of coronary artery lesions was quantified by the synergy between PCI with taxus and cardiac surgery (SYNTAX) score, which was calculated using the online calculator for SYNTAX score.

### Statistical analysis

Continuous variables were expressed as the mean (${\bar{\text{X}}}$) ± standard deviation (SD) in the case of normal distribution, and differences between two groups were determined by two-sided *t*-test. Data were expressed as medians (interquartile ranges, P_25_, and P_75_) in the case of skewed distribution and compared between two groups using the Mann–Whitney test. Categorical variables were presented as counts (percentages) and compared by Chi square test.

Univariate Cox’s proportional hazards regression modelling was performed to identify determinants of ISR in diabetic patients. Baseline variables were selected if they had either a clinically plausible relation with the ISR or appeared to be imbalanced between ISR and non-ISR patients with a *P*-value less than 0.2. The potential variables were entered into multivariate Cox’s proportional hazards regression modelling using the stepwise method (entry, 0.05; removal, 0.05) to determine their independent risk associated with ISR in diabetes. The hazard ratio (HR) and 95% confidence intervals (95% CIs) were calculated to estimate the adjusted risk of ISR in diabetic patients. The predictive value of the Cox’s regression model was evaluated using the area under the receiver operating characteristics curve (AUC).

According to the ROC, the optimal cutoff point of the RLP-C was identified, and patients were divided into 2 groups. Propensity score matching analysis was performed in the two groups with a proportion of 1:1. Log-rank tests were used to compare Kaplan–Meier curves for overall follow-up to assess ISR between the two groups.

Statistical analyses were performed using SPSS software for Windows (version 24.0, SPSS Inc., Chicago, Illinois, USA). A two-sided probability value of < 0.05 was considered statistically significant in all analyses.

## Results

### Baseline clinical and angiographic characteristics (Total population)

**Fig. 1:**
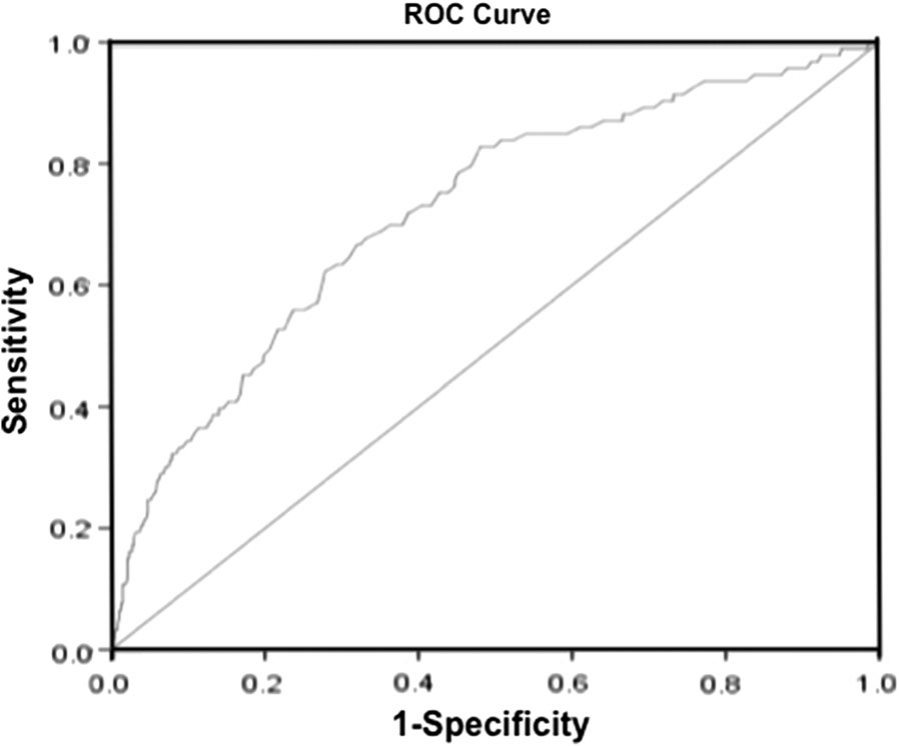
The predictive values of RLP-C level for predicting the risk of ISR. *ROC* receiver operating characteristic, *CI* confidence interval

The baseline clinical and angiographic characteristics of the total population are shown in Table 1. Significant differences were observed between the ISR and non-ISR group in terms of smoking, medical history, TG, HDL-C and RLP-C. After adjusting for other confounding factors in the multivariate Cox’s proportional hazards regression, the RLP-C level was identified as one of the independent predictors associated with ISR in diabetic patients (Table 2). ROC curve analysis indicated that the AUC was 0.722 (95% CI = 0.693–0.751, *P *< 0.001), which showed a good predictive accuracy of RLP-C for the risk of ISR in diabetic patients after baseline PCI (Fig. 1). The baseline RLP-C level at 0.505 mmol/L (19.4 mg/dL) was identified as the optimal cutoff point to predict the risk of ISR with a sensitivity of 82.8% and a specificity of 52.0%.

**Table 1:** Baseline clinical and angiographic characteristics of study population

Characteristics	Total (n = 2312)	ISR (n = 372)	Non-ISR (n = 1940)	*P* values
Age, years	56.21 ± 9.65	55.31 ± 9.67	56.38 ± 9.63	0.050
Male, n (%)	1652 (71.5)	272 (73.1)	1380 (71.1)	0.438
BMI, kg/m^2^	26.45 ± 3.04	26.52 ± 2.99	26.43 ± 3.05	0.618
SBP, mmHg	131.45 ± 17.65	130.40 ± 15.92	131.65 ± 17.96	0.209
DBP, mmHg	77.97 ± 10.23	77.63 ± 10.97	78.04 ± 10.09	0.509
Smoking, n (%)	928 (40.1)	116 (31.2)	812 (41.9)	< 0.001
Drinking, n (%)	360 (15.6)	64 (17.2)	296 (15.3)	0.343
*Medical history, n (%)*				
Hypertension	1568 (67.8)	240 (64.5)	1328 (68.5)	0.136
Hyperlipidaemia	1140 (49.3)	228 (61.3)	912 (47.0)	< 0.001
History of MI	204 (8.8)	24 (6.5)	180 (9.3)	0.078
History of stroke	180 (7.8)	16 (4.3)	164 (8.5)	0.006
Family history of CAD	208 (17.6)	68 (18.3)	340 (17.5)	0.727
Symptom for CAG	1354 (58.6)	234 (62.9)	1120 (57.7)	0.064
*Laboratory results*				
TG, mmol/L	2.22 ± 1.54	2.62 ± 2.16	2.14 ± 1.37	< 0.001
TC, mmol/L	4.43 ± 1.07	4.81 ± 1.12	4.35 ± 1.04	< 0.001
LDL-C, mmol/L	2.77 ± 0.90	2.81 ± 0.87	2.77 ± 0.94	0.348
HDL-C, mmol/L	1.00 ± 0.26	1.04 ± 0.97	0.57 ± 0.44	< 0.001
RLP-C, mmol/L	0.65 ± 0.59	0.94 ± 1.00	0.59 ± 0.44	< 0.001
FBG, mmol/L	7.69 ± 2.50	7.64 ± 2.20	7.71 ± 2.55	0.591
HbA1c, %	7.35 ± 1.30	7.41 ± 1.21	7.34 ± 1.31	0.283
hs-CRP, mg/L	4.72 ± 6.98	4.60 ± 5.93	4.75 ± 7.16	0.662
Creatinine, μmol/L	77.35 ± 19.15	77.10 ± 18.23	77.40 ± 19.33	0.782
GFR, ml/min	113.70 ± 316.21	184.46 ± 782.91	100.13 ± 27.43	0.038
UA, μmol/L	335.49 ± 107.15	332.01 ± 129.22	336.16 ± 102.40	0.560
LVEF, %	61.83 ± 8.14	62.15 ± 7.37	61.76 ± 8.28	0.363
*Medical treatment, n (%)*				
Statins	2164 (93.6)	340 (91.4)	1824 (94.0)	0.058
Aspirin	2272 (98.3)	364 (97.8)	1908 (98.4)	0.497
β-Blocker	1776 (76.8)	284 (76.3)	1492 (76.9)	0.814
Clopidogrel	2292 (99.1)	364 (97.8)	1928 (99.4)	0.003
Insulin	556 (24.0)	76 (20.4)	480 (24.7)	0.075
ACEI	700 (30.3)	92 (24.7)	608 (31.3)	0.011
ARB	548 (23.7)	80 (21.5)	468 (24.2)	0.268
*Number of target vessels*				< 0.001
One, n (%)	792 (34.3)	124 (33.3)	668 (34.4)	
Two, n (%)	948 (41.0)	112 (30.1)	836 (43.1)	
Three, n (%)	564 (24.4)	128 (34.4)	436 (22.5)	
*Target vessels*				
LM, n (%)	56 (2.4)	8 (2.2)	48 (2.5)	0.710
LAD, n (%)	1316 (56.9)	244 (65.6)	1072 (55.3)	< 0.001
LCX, n (%)	724 (31.1)	140 (37.6)	584 (30.1)	0.004
RCA, n (%)	884 (38.2)	160 (43.0)	724 (37.3)	0.039
SYNTAX score	12.07 ± 6.98	14.08 ± 7.55	11.68 ± 6.79	< 0.001
Minimal stent diameter, mm	2.95 ± 0.46	2.93 ± 0.41	2.95 ± 0.47	0.460
Stent length, mm	22.02 ± 6.60	22.63 ± 5.82	21.90 ± 6.75	0.035

*ISR* in-stent restenosis, *BMI* body mass index, *SBP* systolic blood pressure, *DBP* diastolic blood pressure, *MI* myocardial infarction, *CAD* coronary artery disease, *TG* triglyceride, *TC* total cholesterol, *LDL-C* low-density lipoprotein cholesterol, *HDL-C* high-density lipoprotein cholesterol, *RLP-C* remnant-like particle cholesterol, *FBG* fasting blood glucose, *hs-CRP* high-sensitivity C-reactive protein, *GFR* glomerular filtration rate, *UA* uric acid, *LVEF* left ventricular ejection fraction, *ACEI* angiotensin converting enzyme inhibitor, *ARB* angiotensin receptor blocker, *ISR* in-stent restenosis, *LM* left main, *LAD* left anterior descending, *LCX* left circumflex artery, *RCA* right coronary artery, *SYNTAX* synergy between PCI with taxus and cardiac surgery

**Table 2:** Independent predictors of ISR in patients with DM after baseline PCI

Variables	HR	95% CI	*P* values
Model 1	1.609	1.478–1.735	< 0.001
Model 2	2.857	2.324–3.511	< 0.001
Model 3	2.763	2.216–3.446	< 0.001

Model 1: age, male, smoking, hyperlipidaemia, history of stroke

Model 2: model 1 + TG, TC, HDL-C, GFR, clopidogrel, ACEI

Model 3: Model 2 + number of target vessels, LAD, LCX, RCA, SYNTAX score, stent length

Continuous variables were expressed as the mean (${\bar{\text{X}}}$) ± standard deviation (SD) in the case of normal distribution and compared between two groups by the independent samples *t*-test. Data were expressed as medians (interquartile ranges) in the case of skewed distribution and compared using the Mann–Whitney *U*-test. Categorical variables are presented as counts (percentages) and compared by the Chi square test.

### Baseline clinical characteristics, angiographic characteristics and Kaplan–Meier curves (propensity score matching population)

**Fig. 2:**
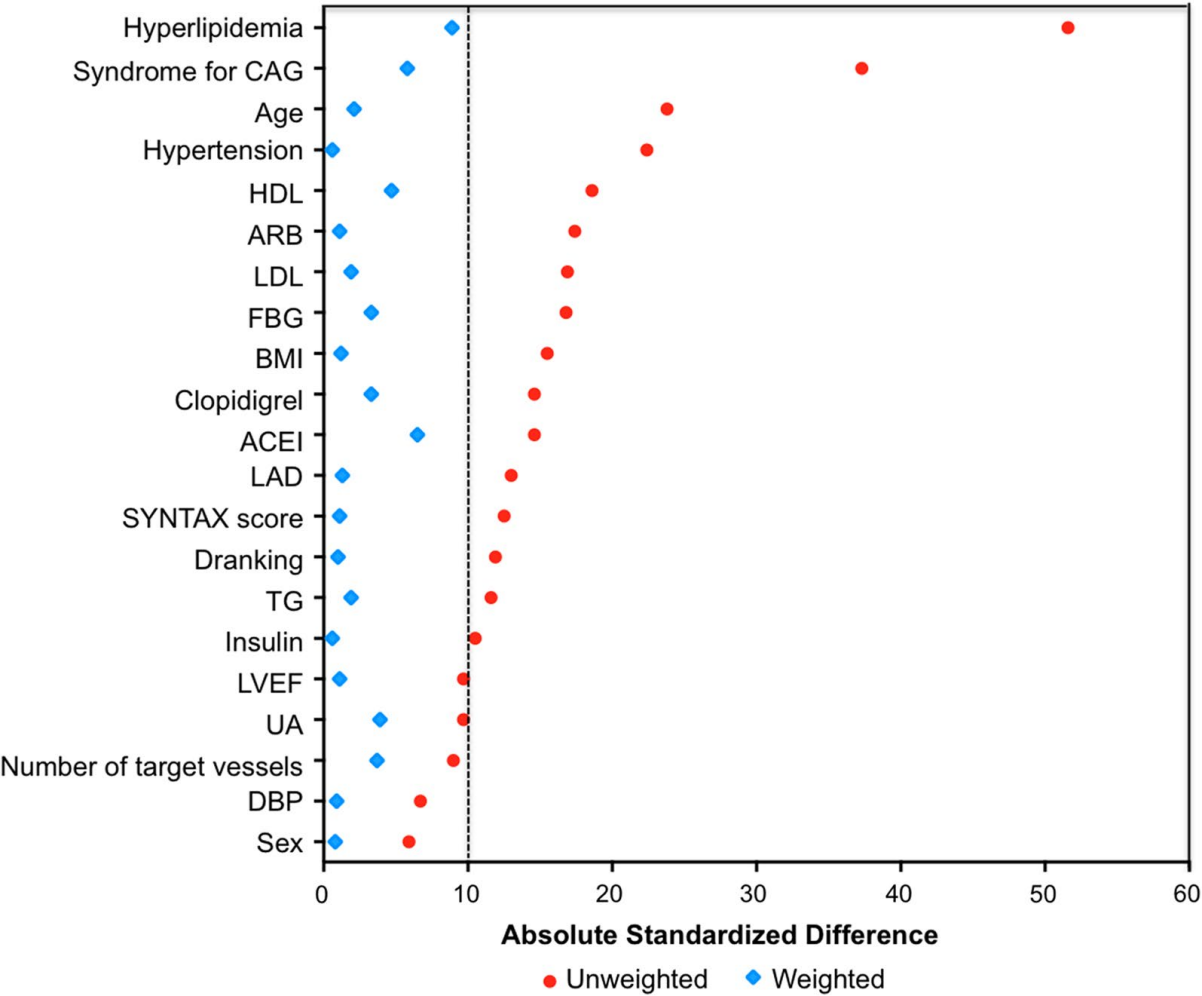
Absolute standardized differences in unweighted and propensity score-weighted data sensitivity analyses. Given the difference in baseline variables between high RLP-C and low RLP-C groups, a propensity score–based method was performed to balance baselines of the two groups. Importantly, after propensity score matching, all between-group standardized differences were < 10. *CAG* coronary artery angiography, *HDL-C* high-density lipoprotein cholesterol, *ARB* angiotensin receptor blocker, *LDL-C* low-density lipoprotein cholesterol, *FBG* fasting blood glucose, *ACEI* angiotensin converting enzyme inhibitor, *LAD* left anterior descending, *SYNTAX* synergy between PCI with taxus and cardiac surgery, *TG* triglyceride, *LVEF* left ventricular ejection fraction, *UA* uric acid, *DBP* diastolic blood pressure

After propensity score matching analysis, a total of 762 pairs of matched patients were created. The C statistics for the propensity score model was 0.01. The baseline clinical characteristics before and after propensity score-matched analysis are shown in Fig. 2. No significant differences were observed between the low and high RLP groups after propensity score–matched analysis in terms of age, gender, BMI, medical history, duration of diabetes mellitus, HbA1c, fasting glycaemia, drug use, other biomarkers, angiographic characteristics and procedural characteristics.

**Fig. 3:**
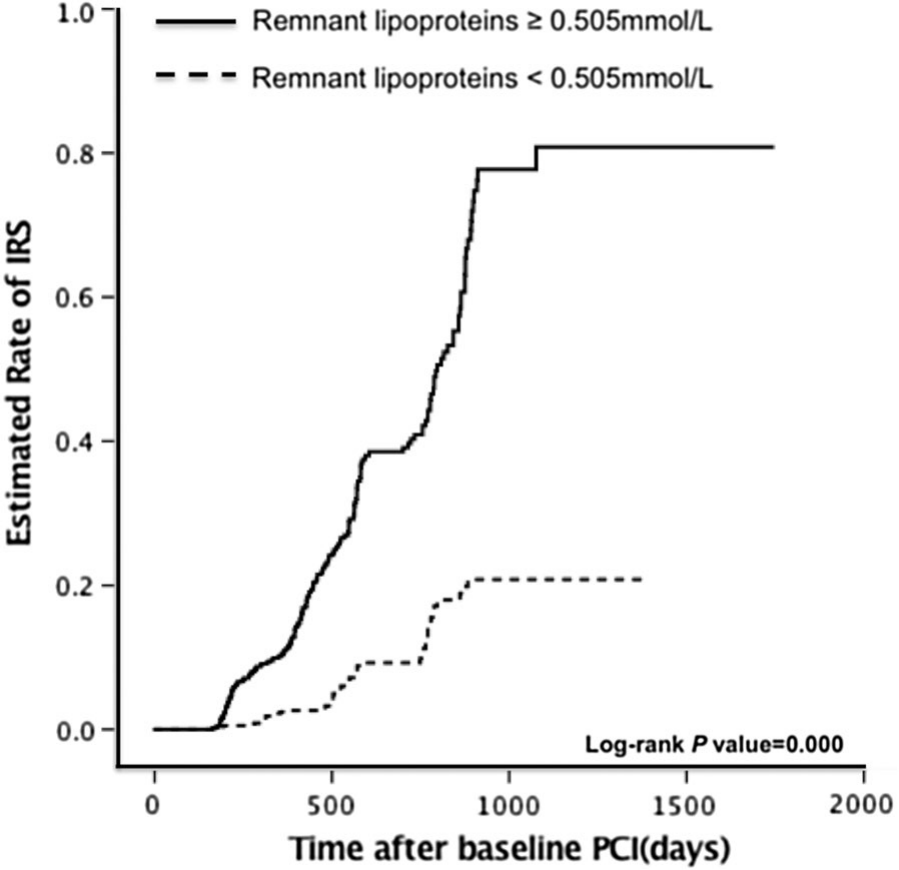
Kaplan–Meier curves for estimated cumulative rate of ISR. *ISR* in-stent restenosis, *PCI* percutaneous coronary intervention

Log-rank tests were used to compare Kaplan–Meier curves for overall follow-up to assess ISR between the two groups (Fig. 3).

## Discussion

### Main findings

The present observational cohort study from a high volume cardiovascular centre in China revealed potential atherosclerosis resulting from remnant lipoproteins in the occurrence and development of in-stent restenosis in diabetic patients. The major findings were as follows: (1) the presence of remnant-like particle cholesterol is an independent risk factor for in-stent restenosis in diabetic patients; and (2) diabetic patients with high RLP-C levels (≥ 0.505 mmol/L) have greater risk for in-stent restenosis compared to patients with low RLP-C levels.

### Abnormal lipid metabolism and atherosclerosis in DM

It is well known that LDL-C is the major risk factor for atherosclerosis and CVD [11, 15]. However, several recent meta-analyses have indicated that a high residual risk of CVD remains even in patients whose LDL-C levels reach the treatment target after statin treatment [16, 17]. Additionally, current dyslipidaemia guidelines recommend non-HDL-C as the primary target of lipid-lowering therapy [10], including VLDL-C, which is the major component of RLP-C during fasting.

However, diabetic patients have dyslipidaemia characterized by high levels of RLP-C [7] but normal levels of LDL-C. Existing research has shown that increased remnant lipoprotein level is a risk factor for ischaemic heart disease. With an empty stomach, an increase of 1 mmol/L residual lipoprotein increases ischaemic heart disease risk by 2.8 times [18]. Recently, prospective studies tracking coronary events in diabetic patients have shown that remnant-like particle cholesterol are the most important independent risk factors of coronary artery disease and can predict coronary events [19, 20].

Both in vitro and animal experiments have confirmed that the formation of atherosclerosis induced by increases in remnant lipoproteins is similar to the formation of atherosclerosis caused by the accumulation of lipid in the arterial wall induced by increases in low-density lipoprotein. Many studies have confirmed that the mechanism of atherosclerosis induced by remnant lipoproteins mainly manifests in the following aspects: (1) induction of proliferation of smooth muscle cells but no involvement in oxidative stress [21]; (2) induction of apoptosis in endothelial cells [22]; (3) induction of mononuclear/macrophage migration in endothelial cells [23]; (4) induction of McP-1 expression in umbilical venous blood endothelial cells as well as early growth response factor-1 (egr-1) mRNA and protein expression in vascular smooth muscle cells, which induces the occurrence of inflammation [24]; and (5) induction of elevated levels of other atherogenic lipoproteins.

### Contrast and enlightenment

China has over 92.4 million diabetic patients (9.7% of the adult population), which ranks at the top with DM patient numbers and higher diabetes-related burden than other countries [25]. A single-centre study from Fuwai Hospital of China [26] reported that second-generation drug-eluting stents have reliable efficacy and safety in diabetic and non-diabetic patients. In the subgroup analysis of diabetic patients, the risk factors associated with target lesion revascularization included current smoker, a history of coronary heart disease, and old myocardial infarction. In the baseline data of this study, only the history of hyperlipidaemia was associated with blood lipids, and the specific content of each component of the relevant blood lipids was not included, especially remnant-like particle cholesterol (possibly due to the large difference in blood lipid spectrum between diabetic and non-diabetic people). All patients included in the present study were treated with second generation drug-eluting stents. The baseline contained the contents of various blood lipid components. Propensity score matching found that remnant lipoproteins played an important role in in-stent restenosis, which supplemented findings from previous studies. In the present study, the TG level was higher in the in-stent restenosis group, which was attributed to the TG-rich remnant lipoproteins [8]. Therefore, TG increased with the increase of remnant lipoproteins. Patients were divided into two groups according to the remnant cholesterol level, and the baseline data of the two groups were matched with propensity scores. After matching, there was no statistically significant difference in TG between the two groups, excluding the influence of TG on the results, which constituted a two-way verification process with the previous multi-factor analysis.

Previous studies [27] have proven that the association between disorders of TG metabolism and remnant-like particle cholesterol may account for the risk of CAD in diabetic patients. According to the relationship between the level of remnant-like particle cholesterol and the degree of in-stent restenosis confirmed by coronary angiography, the present study further confirmed that the remnant-like particle cholesterol rich in triglycerides play an important role in atherosclerosis in coronary heart disease patients with diabetes mellitus. Therefore, it is important to strengthen the management of remnant-like particle cholesterol in diabetic population in addition to the control of LDL-C levels required by the guidelines.

Statins play an irreplaceable role in secondary prevention of coronary heart disease. However, T2DM patients on statin therapy presenting increased levels of cholesterol remnants and triglycerides are prone to slight decreases in left ventricular systolic function [28], which severely affects the prognosis of diabetic patients with coronary heart disease. Therefore, in the secondary prevention of coronary heart disease in diabetic patients, it is important to use statins to reduce LDL-C while monitoring remnant-like particle cholesterol. Previous studies have confirmed that empagliflozin [29] and pemafibrate [30] lower remnant-like particle cholesterol, indicating that they have a good curative effect and are recommended to delay the progression of coronary atherosclerosis.

### Limitations

Some limitations and strengths of the present study need to be acknowledged. First, the study was only a single-centre study, which may weaken the statistical power of the conclusions. Second, propensity score matching provides yields weaker evidence than randomized controlled trials.

## Conclusions

In conclusion, the present study further emphasized the importance of atherogenic lipids and remnant particles in cardiovascular pathology (such as in-stent restenosis), especially in diabetic patients. Physicians should take measures to lower the level of remnant-like particle cholesterol to < 0.505 mmol/L to better prevent in-stent restenosis in diabetic patients.

## Abbreviations

ACEI: angiotensin converting enzyme inhibitor
ARB: angiotensin receptor blocker
BMI: body mass index
BP–DESs: biodegradable polymer drug-eluting stent
CAD: coronary artery disease
CRF: case report form
DBP: diastolic blood pressure
DM: diabetes mellitus
ECG: electrocardiograph
FBG: fasting blood glucose
G2-DESs: second–generation drug-eluting stent
GFR: glomerular filtration rate
HDL-C: high density lipoprotein cholesterol
HPLC: high performance liquid chromatograph
hs-CRP: high-sensitivity C-reactive protein
ISR: in-stent restenosis
LAD: left anterior descending
LCX: left circumflex artery
LDL-C: low density lipoprotein cholesterol
LM: left main
LVEF: left ventricular ejection fraction
MI: myocardial infraction
MVD: multivessel disease
Non-HDL-C: non-high density lipoprotein cholesterol
PCI: percutaneous coronary intervention
RCA: right coronary artery
ROC: receiver operating characteristics
RLP-C: remnant-like particle cholesterol
SBP: systolic blood pressure
SYNTAX: synergy between PCI with taxus and cardiac surgery
TC: total cholesterol
TG: triglyceride
UA: uric acid
VLDL-C: very low density lipoprotein cholesterol
